# Transcriptomic and proteomic analysis of a compatible tomato-aphid interaction reveals a predominant salicylic acid-dependent plant response

**DOI:** 10.1186/1471-2164-14-515

**Published:** 2013-07-29

**Authors:** Valentina Coppola, Mariangela Coppola, Mariapina Rocco, Maria Cristina Digilio, Chiara D’Ambrosio, Giovanni Renzone, Rosanna Martinelli, Andrea Scaloni, Francesco Pennacchio, Rosa Rao, Giandomenico Corrado

**Affiliations:** 1 Dipartimento di Agraria, Università degli Studi di Napoli Federico II, 80055 Portici, NA, Italy; 2 Università degli Studi del Sannio, 82100 Benevento, Italy; 3 Istituto per il Sistema Produzione Animale in Ambiente Mediterraneo-CNR, 80147 Napoli, Italy; 4 CEINGE, 80145 Napoli, Italy

**Keywords:** *Solanum lycopersicum*, *Macrosiphum euphorbiae*, Plant-insect interactions, Defense, Salicylic acid, Jasmonic acid

## Abstract

**Background:**

Aphids are among the most destructive pests in temperate climates, causing significant damage on several crops including tomato. We carried out a transcriptomic and proteomic study to get insights into the molecular mechanisms and dynamics of the tomato response to the *Macrosyphum euphorbiae* aphid.

**Results:**

The time course analysis of aphid infestation indicated a complex, dynamic pattern of gene expression. Several biological functions were affected and genes related to the stress and defence response were the most represented. The Gene Ontology categories of the differentially expressed genes (899) and identified proteins (57) indicated that the tomato response is characterized by an increased oxidative stress accompanied by the production of proteins involved in the detoxification of oxygen radicals. Aphids elicit a defense reaction based on the cross-communication of different hormone-related signaling pathways such as those related to the salicylic acid (SA), jasmonic acid (JA), ethylene and brassinosteroids. Among them, the SA-signaling pathway and stress-responsive SA-dependent genes play a dominant role. Furthermore, tomato response is characterized by a reduced accumulation of photosynthetic proteins and a modification of the expression of various cell wall related genes.

**Conclusions:**

Our work allowed a more comprehensive understanding of the signaling events and the defense dynamics of the tomato response to aphids in a compatible interaction and, based on experimental data, a model of the tomato–aphid molecular interaction was proposed. Considering the rapid advancement of tomato genomics, this information will be important for the development of new protection strategies.

## Background

The investigation of plant defense mechanism offers interesting information about genes suitable to control agricultural pests [1]. Studies on crop plants, for which an increasing number of genomic sequencing projects have been completed, are essential to translate the knowledge gained on model species into indications useful to select superior genotypes and to develop more efficient control strategies.

Aphids (Hemiptera: Aphididae) are the largest group of phloem-feeders and among the most destructive insect pests of cultivated plants in temperate regions [2]. These insects have a unique feeding strategy and impose a distinctive stress on plants, being able to directly and indirectly damage crops by removing photoassimilates and introducing viruses. Most aphids feed on contents of vascular tissues by inserting piercing mouthparts (i.e. the stylet) hence, causing a limited mechanical damage. However, aphids have the ability to manipulate host plant physiology and to introduce effectors that alter defense signaling [3,4]. Differently from caterpillars, aphids establish a prolonged interaction with the attacked plant tissue. Currently, little is known on how aphids can feed for an extended period of time from a single sieve element, despite the ability of plants to quickly repair damaged tissues. For all these reasons, it is widely accepted that plant response to phloem-feeding aphids is distinct from that to chewing insects, which crush leaf tissue, and to thrips and spider mites, which ingest the content of individual cells [5,6].

The signs and symptoms of aphid attack can be diverse, and vary according to the plant species (and the tissue attacked), to the aphid species and biotype, and their combination [6]. Therefore, it is likely that host molecular response is specific for a certain plant-aphid interaction. Plant-aphid interaction has been studied mainly at the transcriptional level, while proteomic data are only available for wheat attacked by cereal aphids [7]. Overall, it is believed that aphids trigger in plants responses that overlap with those related to wounding and fungal pathogens [8-10]. Transcriptional profiling pointed out variations related to Reactive Oxygen Species (ROS) generation or scavenging, primary metabolism, cell wall fortification and synthesis of secondary metabolites [10-15]. In different interactions it has been observed that plants activate the jasmonic acid (JA)- and/or salicilic acid (SA)-dependent pathways, which should regulate aphid defense genes through their antagonistic or synergistic cross-communication. For instance, the expression of SA-responsive genes increases substantially following the attack of *Myzus persicae* on aphid-susceptible *Arabidopsis* and celery, and of *Schizaphis graminum* on aphid-susceptible sorghum, while changes in JA-dependent mRNA levels were more limited [9-12]. Moreover, the induction of the SA-pathway in aphid-resistant wheat challenged by *Diuraphis noxia* also supports a predominant role of this molecule in the resistance mechanism [16]. However, gene expression profiling indicated that both SA- and JA-responsive genes were substantially induced in *Arabidopsis* by *Brevicorynae brassicae* or *M*. *persicae* attack [17,18]. These apparent discrepancies may be partly explained considering that in a compatible interaction, phloem-feeders may antagonize the innate plant wound responses to make the plant a more suitable host [5]. Currently, the effect of SA induction on aphid performance in compatible interactions, as well as its antagonism with the JA pathway, have not been fully elucidated (reviewed in [1]).

Towards this aim, we studied at the transcriptional and proteomic level the defense response of tomato plants (*Solanum lycopersicum*) against the potato aphid (*Macrosiphum euphorbiae*), a polyphagous pest of remarkable economic importance [19]. In tomato, symptoms include mild leaf curling, chlorosis and necrosis, resulting in defoliation and significant yield loss when pest population density is high [19]. In the present work, a time-course transcriptomic analysis based on microarrays was carried out to investigate tomato responses during a compatible interaction. In addition, to achieve a more detailed understanding of the tomato response, we performed a proteomic analysis by 2-D electrophoresis combined with MS technology. Our work provided the first combined analysis of the tomato-aphids molecular interaction.

## Results

### Expression profiling of tomato genes responsive to *M*. *euphorbiae* feeding

**Figure 1:**
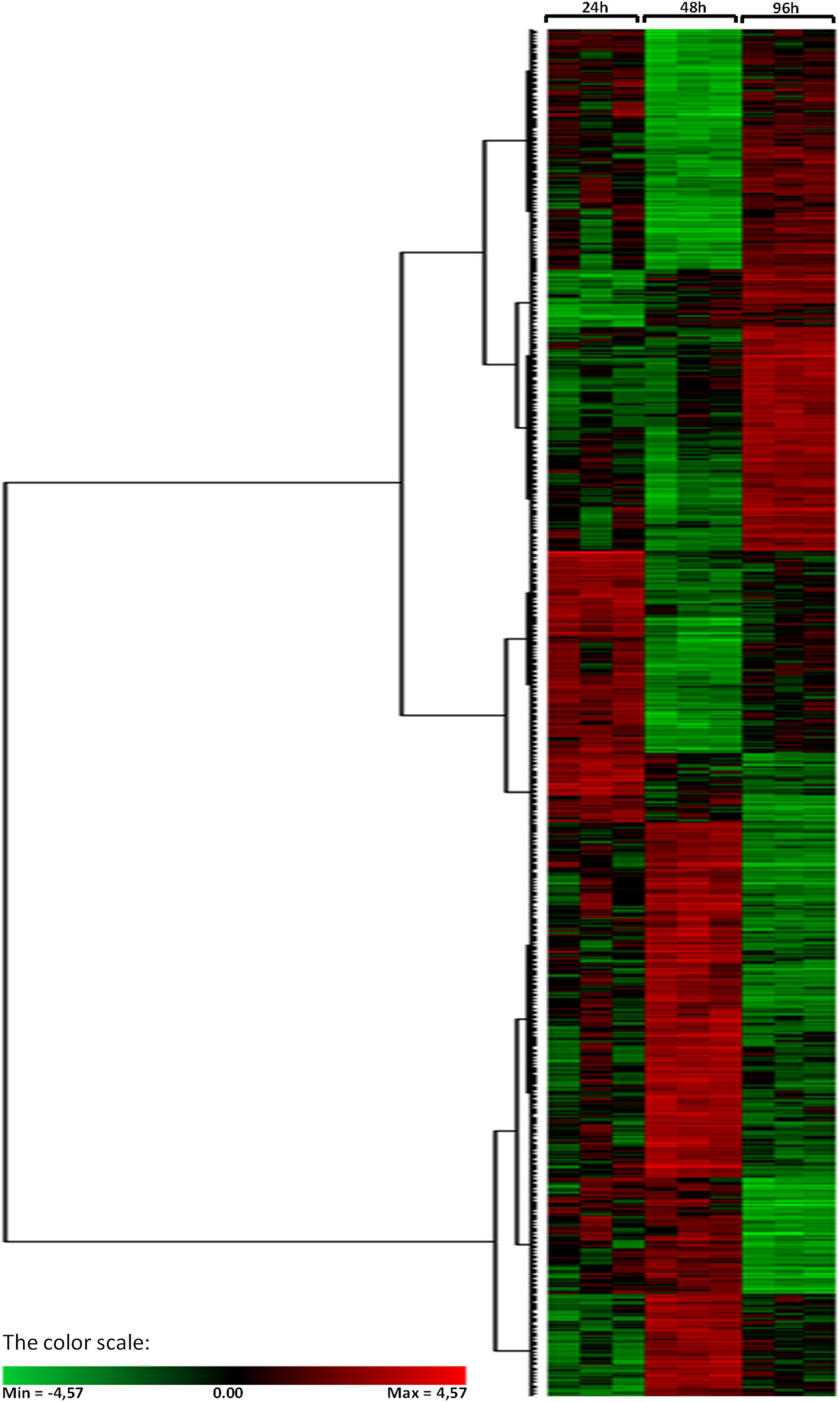
**Hierarchical cluster of all differentially expressed probes.** Distances were calculated using the Pearson similarity and agglomeration was performed according to the Ward's minimum variance algorithm. The heat-map diagram shows the relative expression level at the three time points (24, 48 and 96 hours post infection). Gradation from red to green represents strong up-regulation to strong down-regulation on a log scale. In each time point, each colored column represents a single biological replicate.

**Figure 2:**
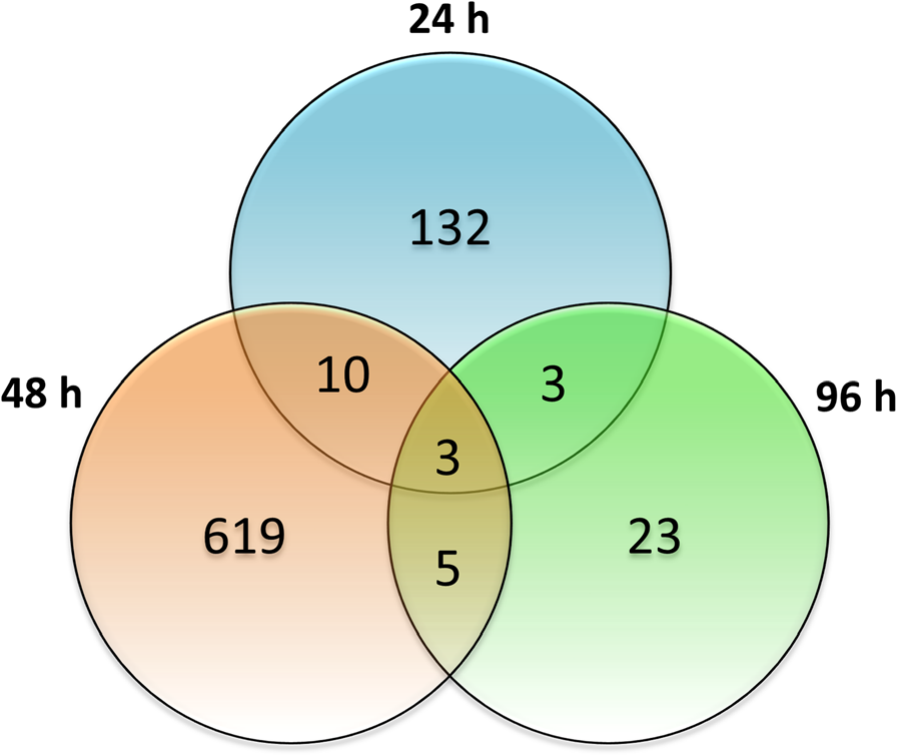
Venn diagram showing the number of overlapping and non overlapping differentially expressed tomato genes in the comparisons between the three sampling times.

To profile the variation in gene expression in tomato following the establishment of a compatible interaction with the *M*. *euphorbiae*, we analysed plants 24, 48 and 96 hours after infestation. In our data analysis, three filtering criteria were used to identify differentially expressed genes: a two-fold change in transcript levels between unifested and infested plants, a *p* <0.05 and a significant match between the oligonucleotide probe and a tomato gene. Taking into account the three time points, 999 annotated probes were significantly differentially expressed (Table 1). All differentially expressed probes were grouped according to the similarity of their expression profiles at the three data-points by cluster analysis. The dendrogram indicated that, as expected, the three biological replicates for each time point assort together, showing a good congruence (Figure 1). Moreover, the heat-map illustrates a weak linkage among the three conditions, with the most intense transcriptional response at 48 h. Cluster analysis identified groups of similarly behaving transcripts that have different expression trends, highlighting a relevant dynamism of gene expression in tomato. The recent release of the tomato genome sequence allowed us to map the microarray probes on the genome sequence, thus providing the opportunity of a more accurate functional annotation of the array. A similarity analysis, performed against SGN Tomato Unigene database for the differentially expressed 999 probes, identified 819 genes. Specifically, the data indicated that at 24 h after infestation, 148 genes (72 up and 76 down) were significantly affected by aphid infestation. The number of responsive genes at 48 h was 637 (320 genes up and 317 down), while at 96 h, 34 genes (17 up and 17 down) were differential expressed. The complete list of differentially expressed genes, including their expression levels in all three time points, is accessible as supplementary material (Additional file 1: Table S2, S3, S4). The Venn diagram (Figure 2) shows the intersections between the differentially expressed genes at the three time points. For all combinations, the overlap was limited, and only three genes were significantly affected throughout the whole time course (Table 2), indicating that the induction of most response genes is transient [20]. It is therefore noteworthy that two genes encode transcription factors belonging to the WRKY family, important regulators of SA-dependent defense responses. Specifically, the WRKY6 gene codes for a protein that in *Arabidopsis thaliana* has the highest similarity to the AtWRKY70 (identity: 41%; similarity: 58%; e-value: 1e-36), considered its orthologue [21]. This is strengthened by the presence in the WRKY6 promoter region of the core binding consensus sequence for the AtMYB44, which was recently described as a transcriptional activator in *Arabidopsis*[22]. The tomato WRKY46 protein is most similar to AtWRKY40 (identity: 43%; similarity: 54%; e-value: 2e-54). The AtWRKY40 gene is associated with pathogen response and it was also induced by the aphid *B*. *brassicae*[14]. The third gene codes for a GDSL esterase/lipase. GDSL-lipase belongs to a subfamily of lipolytic enzymes which appear to be primarily involved in the regulation of plant development and morphogenesis [23]. Some GDSL-lipase genes have been involved in plant defense because of their induction by SA and pathogens [24-26].

**Table 1:** **Tomato probes (999) differentially expressed in response to *M*. *euphorbiae *feeding at each sampling time**

Time (hpi)*	**Number of probes**		
	**Up-regulated**	**Down-regulated**	**Total**
24	87	95	182
48	391	377	768
96	23	26	49

* hpi: hours post infestation.

**Table 2:** **Shared genes differentially expressed in response to *M*. *euphorbiae *feeding at the three sampling times**

**Fold change**			**SGN Unigene**	**Description**	GO annotation*
**24 h**	**48 h**	**96 h**			
3.5	8.5	3.6	Solyc09g015770.2.1	WRKY transcription factor 6	P: regulation of transcription;
					F: transcription factor activity; C: transcription factor complex
10.7	124.6	31.7	Solyc08g067340.2.1	WRKY transcription factor 46	P: regulation of transcription;
					F: transcription factor activity; C: transcription factor complex
3.13	−2.49	2.22	Solyc11g006250.1.1	GDSL esterase/lipase	P: lipid metabolic process;
					F: hydrolase activity;
					C: cytoplasmic membrane-bounded vesicle

* The GO term associations for the gene product were retrieved and selected from the best blast matches at the AmiGO website (http://amigo.geneontology.org). P: biological process; F: molecular function; C: cellular component.

### Dynamics of the tomato response to aphids

To underline the biological objective to which the differentially expressed genes contribute, we used the Blast2GO tool to provide Gene Ontology (GO) terms association. The differentially expressed genes were distributed in eleven categories, namely “cell maintenance”, “transcription”, “cell wall modification”, “stress and defense response”, “signal transduction”, “photosynthesis”, “primary metabolism”, “secondary metabolism”, “protein metabolism”, “transport” and “unknown function”.

**Figure 3:**
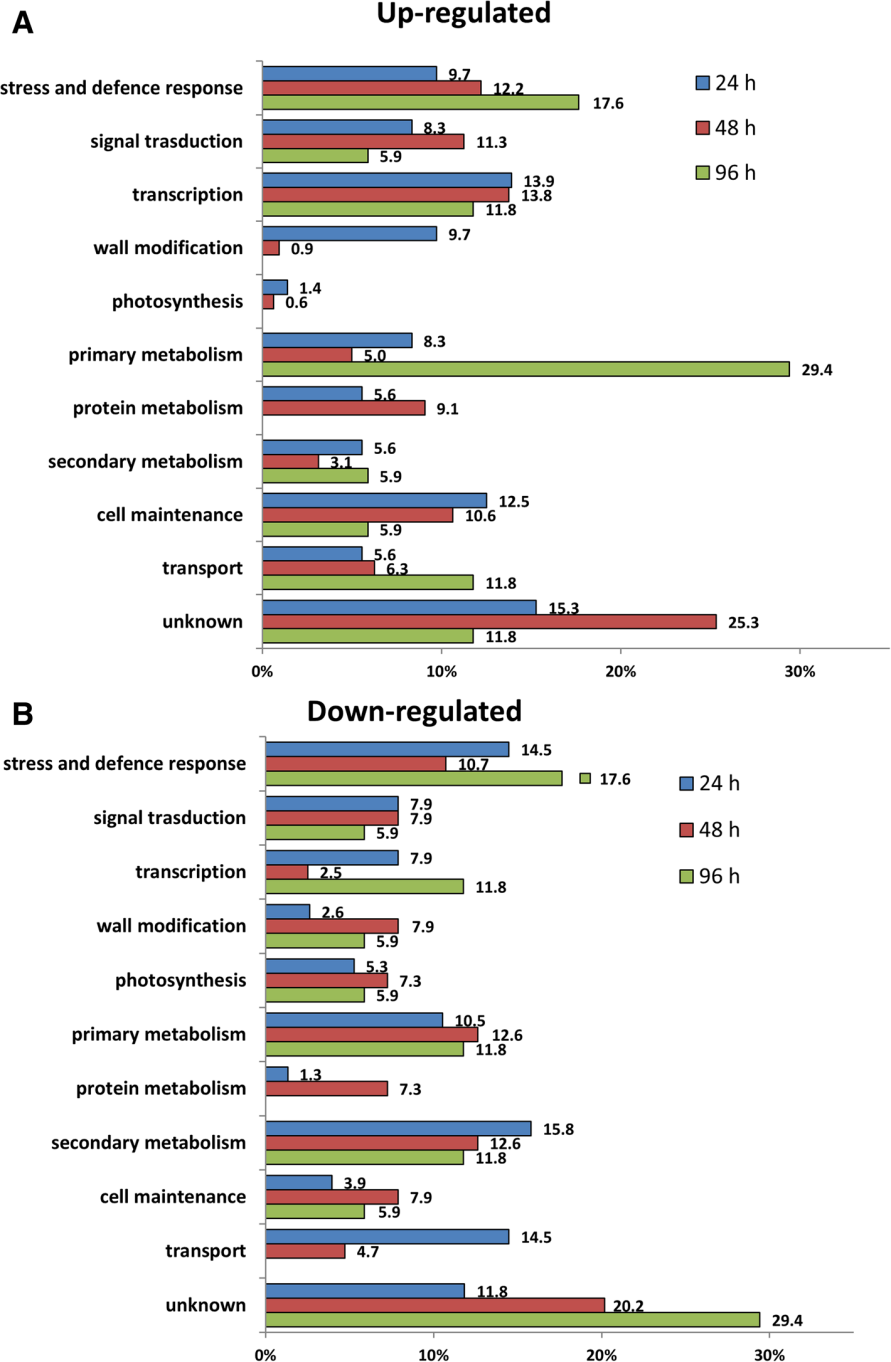
**Functional overview of the differentially expressed genes.** Functional analysis of the up-regulated **(A)** and down-regulated **(B)** tomato genes following *M*. *euphorbiae* attack at the three harvest times. Differentially expressed genes were assigned to categories according to GO biological process terms. The X-axis indicates the percentage of the Unigenes in each category out of the total number of differentially-expressed genes for each harvest time.

The investigation of the tomato transcriptome following aphid attack highlighted the activation of a wide and complex response, as the transcriptional reconfiguration involved a broad range of biological processes in a different way. Since the absolute number of differentially expressed genes was different at the three time-points, Figure 3 shows the changes of *M*. *euphorbiae*-induced responses as percentage. Overall, the most relevant differences were in the category “stress and defense response”, followed by “primary metabolism” and “transcription”. The “signal transduction” and “protein metabolism” processes peaked at 48 h. A maximum variation of up-regulated genes at 96 h was found for “primary metabolism”, “transport” and “stress defense and response”. For the biological process “transport” the proportion of down-regulated genes increased in time, but considerably increased at 96 h for “stress defense and response”. This biological process showed the maximum variation of up and down regulated genes at 96 h. With the progress of aphid infestation, the rate of up regulated genes involved in “photosynthesis”, “cell maintenance” and “cell wall modification” gradually decreased in time. For these processes the percentage of downregulated genes peaked at 48 h.

**Figure 4:**
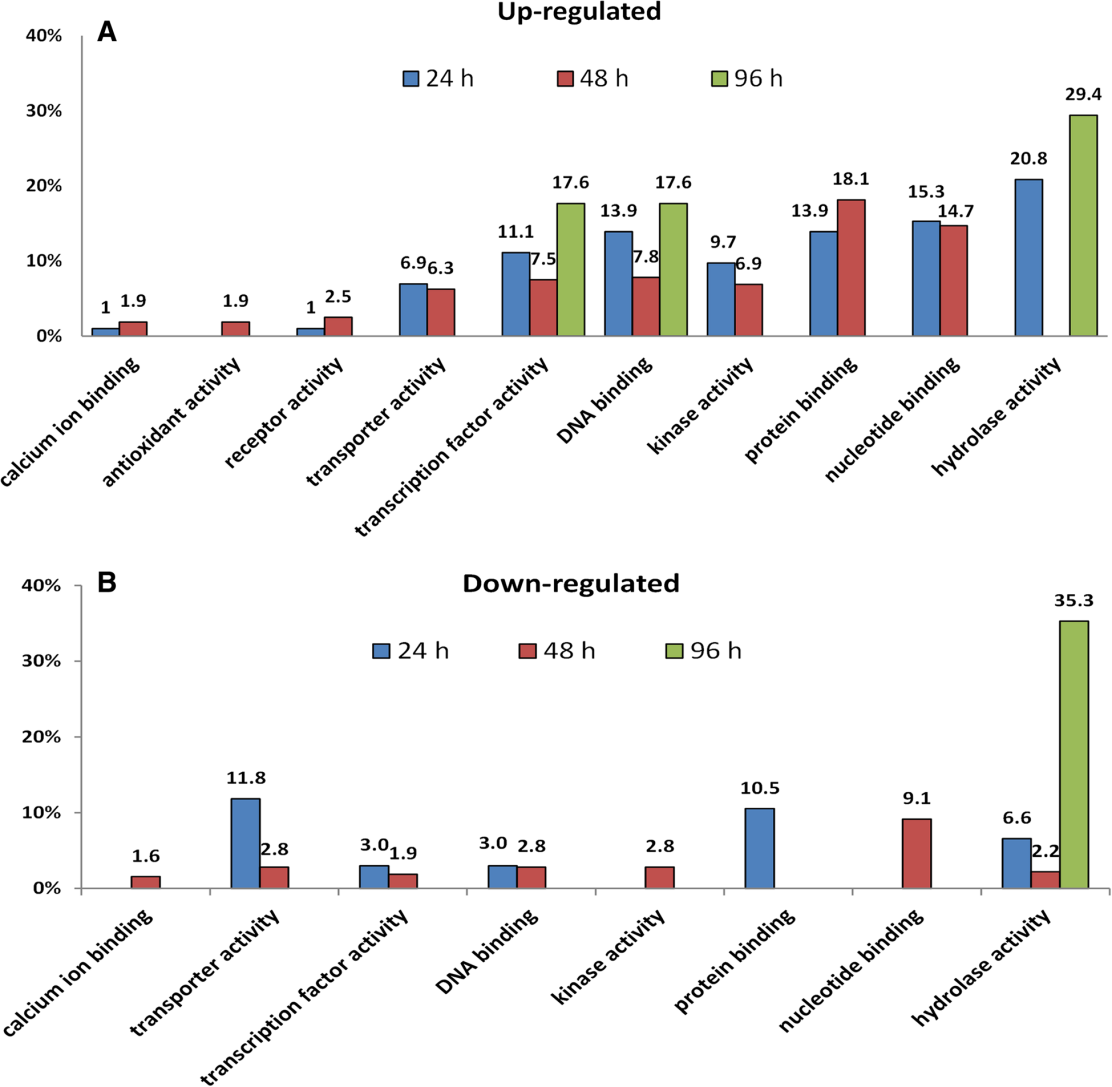
**Molecular functions of the differentially expressed gene.** Distribution of molecular function terms for the up-regulated **(A)** and down-regulated **(B)** tomato genes following *M*. *euphorbiae* damage at the three harvest times, according to GO classification. The X-axis indicates percentage of the Unigenes in each category out of the total number of differentially-expressed genes for each harvest time.

The classification of differentially expressed genes according to GO molecular function terms, indicated that “hydrolase activity” was the most frequent in the first (24 h) and last time (96 h) of infestation (Figure 4). Interestingly, “calcium ion binding”, “kinase activity” and “receptor activity” were the molecular functions present only at 24 h and 48 h, implying that the differentially expressed genes of these categories relate with the perception of aphid feeding and the mounting of a defense response. The molecular function “antioxidant activity” was activated only at 48 h.

Overall, both the number of genes and the categories of the differentially expressed genes indicated that transcriptional re-programming is a key process of a tomato defense towards *M*. *euphorbiae*. The Gene Ontology analysis of the response dynamics showed an active, and for some processes coordinated, alteration of several biological functions. Aphid infestation mainly affects genes involved in stress response and progressively inhibits the expression of genes involved in photosynthesis and cell maintenance. However, in absolute terms the composite tomato response to aphids relies largely on genes that do not code for products directly involved in insect resistance.

### Tomato transcriptional response to aphids

The aphid response genes were grouped according to their functions. For sake of simplicity, genes that participate in more than one biological processes are presented only once considering their prevalent role.

#### Stress and defense response

This functional category comprised genes related to defense against abiotic or biotic stress, including those involved in the response to oxidative stress. Throughout the three time points, a total of 97 genes were differentially expressed (Additional file 1: Table S2, S3, S4), making this category the most represented biological process. Among the overexpressed genes, fourteen were associated to oxidative stress, including, for instance, a gene coding for Respiratory Burst Oxidase-Like Protein (RBOHD). The majority of those genes (9) encode enzymes linked to the oxidative burst, such as four peroxidases, three glutathione S-transferases and two glutaredoxins. Peroxidases code for ROS-detoxifying enzymes but they are also involved in oxidative signal transduction, regulating the redox and Ca^2+^ homeostasis as well as the expression of defense genes [27]. Similarly, glutaredoxins are also involved in the SA signaling pathway [28], through the reduction of the cytosolic form of NPR1 to its monomeric form [29]. Furthermore, as the ROS damage could be enhanced through the accumulation of other toxic compounds (e.g. reactive aldehydes), we observed at the same time point the induction of genes such as Aldehyde dehydrogenase 1 and Aldo/keto reductase. These play a central role in the detoxification mechanism of toxic aldehydes [30,31], which arise from the breakdown of membrane’s lipids due to ROS.

Genes affected by aphid infestation were also related to the SA- (23 genes) and the JA- (8 genes) dependent pathways. Among the genes associated with the SA, there were chitinases, Pathogenesis Related (PR) proteins, and Nimin2c. The latter showed a transient boost, with the highest level of expression at 48 h (39,45 signal log ratio) and a reduced expression at 96 h of infestation (7,52). Nimin genes belong to small families linked to the SAR response, essentially through the interaction with NPR1 [32,33]. In tobacco, NIMIN2 gene expression is elevated prior to the induction of the PR-1a gene, through transient PR-1 repression before SAR has fully developed [33]. While the majority of SA-related genes were over-expressed, the effect of aphid feeding on the JA-related genes was more complex. Four JA-dependent late genes (e.g.: Polyphenol oxidase (PPO), Threonine deaminase, Type I serine protease inhibitor, Serine carboxypeptidase 1) were downregulated at 48 h. Conversely, genes coding for other antinutritive proteins such as protease inhibitors were induced at 24 h and 48 h. Specifically, genes encoding the Kunitz trypsin inhibitor and the Wound-induced proteinase inhibitor 2 were upregulated at 24 h, while the induction of Proteinase inhibitor I transcripts was observed 48 h post-infestation, along with an Arginase and Leucin aminopeptidase. Finally, nineteen different genes typically linked to abiotic stress response showed differential expression at the three time points and included thirteen genes encoding heat shock and DNAJ chaperone proteins. These proteins play key roles in buffering physiological and developmental variations by acting at multiple levels to maintain homeostasis and or protein stability during stress [34].

The gene expression analysis indicated that the transcriptional response to the accumulation and subsequent detoxification of ROS is taking place mainly at 48 h following infestation. Furthermore, aphid attack activates responses most similar to salicylate-mediated gene induction, although the expression of some jasmonate-related genes is also increased.

#### Signaling related genes

The signaling response in plants requires a variety of messengers and thus, seventy-six genes related to signal transduction were differentially expressed in infested tomato. These included among others, the LRR receptor-like serine/threonine-protein kinase, Serine/threonine-protein kinase receptor, S-locus receptor kinase and TIR-NBS-LRR resistance protein (Additional file 1: Table S2, S3, S4). In the first and second time point, 27 genes coding for kinase receptors were affected, whereas the induction of members of this class of genes was not observed at 96 h. This is consistent with a process of aphid recognition and response that takes place during the first phases of the attack. Among these putative cell surface receptors, a serine-threonine protein and a lectin were the most expressed at 24 h and 48 h, respectively. Moreover, twelve genes associated with signaling transduction of plant hormones (i.e.: Et, JA, ABA, brassinosteroids and auxins) were differentially expressed at 48 h. The intracellular concentration of calcium, one of most important ubiquitous second messengers, usually increases in response to the biotic or abiotic stress. While at 24 h two genes associated to calcium homeostasis showed a down regulation, at 48 h a significant up-regulation was observed for transcripts encoding calmodulins, calmodulin-binding proteins, calcium-dependent protein kinases and calcium-binding calreticulins.

Overall, the transcriptional data indicated that tomato response to *M*. *euphorbiae* is based on the concurrent contribution of different cellular signals.

#### Transcription related genes

Seventy-two differentially expressed genes were annotated as involved in the regulation of transcription (Additional file 1: Table S2, S3, S4). Compared to other categories, at both 24 and 48 h the number of overexpressed genes was much larger than the number of downregulated genes. The most abundant class of upregulated genes at 48 h was the one coding for Pentatricopeptide repeat-containing (PPR) proteins. The PPR is a degenerate 35 amino acid motif that occurs in multiple tandem copies in members of a recently recognized eukaryotic gene family, which is relatively small in both animal and fungal kingdom but largely expanded in plants [35,36]. Although the role of the hundreds of PPR family members in plants has not been clarified, these proteins are usually associated to RNA editing. It is likely that the high number of differentially expressed sequences we found is related to their predicted abundance in the tomato genome, although their simultaneous up regulation may be suggestive of a more rapid RNA decay in organelles under stress conditions. Another class of genes that were only significantly overexpressed is the WRKY family. The two genes upregulated during the whole infestation belong to the family of WRKY transcription factors, which have been long implicated in the regulation of transcriptional response to pathogens. Other up regulated transcription factors involved in plant response to stress were those related to ethylene metabolism, such as the Ethylene-responsive transcription factor 2, Ethylene responsive transcription factor 1b and AP2-like ethylene-responsive transcription factors. However, one gene encoding for Et-regulated transcription factors 1a was repressed at 96 h. Other types of transcription factors implicated in aphid response are members of the RING finger and Zinc finger Family Protein, which play a central role in different biologic process such as pathogen defense response. Different classes of genes involved in transcriptional regulation, including some associated with biotic stress response, were induced by the *M*. *euphorbiae*.

#### Cell wall modification related genes

Following *M*. *euphorbiae* attack, tomato plants modulate also the expression of genes involved in cell wall modification. A total of 43 genes were differentially expressed on the three harvest dates (Additional file 1: Table S2, S3, S4). While at the first time of analysis the majority of the differentially expressed genes were up-regulated, at 48 h only three out of 28 genes were overexpressed. Differentially expressed genes included those associated with cellulose synthesis, the control of oriented deposition of cellulose microfibrils and cell wall strength (Cellulose synthase and Kinesin motor family proteins, wax biosynthesis CER1), as well as those coding for enzymes degrading pectin (Polygalacturonase, Pectinesterase), hemicellulose (Xyloglucan endotransglucosylase/hydrolase 5) and glucans (Endo-1 4-beta-glucanase, Glucan endo-1 3-beta-glucosidase 3/5). Concurrently, genes involved in expansion of the cellulose matrix were mainly downregulated (Expansin and Extensin-like).

The data indicated that aphid infestation in tomato elicits a cell wall remodeling that could play a role in aphid resistance, further deterring insect probing and feeding on the unattacked host tissue.

#### Photosynthesis and primary metabolism related genes

The other fundamental biological processes in which the differentially expressed genes have been classified were photosynthesis (26 genes) and primary metabolism (77) (Additional file 1: Table S2, S3, S4). The category “photosynthesis” comprise genes coding for proteins involved in photosynthetic electron transport chain and proteins belonging or associated to photosystem I and II complexes. We observed a general down-regulation of the genes associated with the photosynthesis during the whole infestation period, but with the higher number of differentially expressed genes after 48 h. Tomato response included genes involved in the primary metabolism, mainly those related to carbohydrate and lipid metabolism. Genes of these two groups were predominantly down-regulated on all three infestation dates, similarly to those involved in photosynthesis. However at 48 h, eight genes involved in carbohydrate catabolism were also induced. Differences of expression for genes related to protein metabolism, translation, folding, proteolysis and amino acid metabolism (in total, 57 genes) were observed at 24 h and 48 h after aphid infestation (Additional file 1: Table S2, S3, S4). One aspartic proteinase nepenthesin-1 gene was strongly upregulated at 48 h following potato aphid introduction, together with other genes involved in protein turnover (i.e. proteases).

Our data indicated that before the appearance of signs of chlorosis, the progression of aphid attack mostly represses photosynthesis-related genes in leaf as well as those involved in primary metabolism.

#### Cell maintenance, transport and secondary metabolism related genes

Seventy-three genes involved in cell maintenance showed differential expression following aphid infestation (Additional file 1: Table S2, S3, S4). This category grouped genes associated with processes that preserve the cell or its components in a stable functional or structural state, such as genes coding for proteins implicated in cell cycle, cellular component organization, cell differentiation, and nucleotide and nucleic acid metabolic processes. In addition, *M*. *euphorbiae* induced 52 genes encoding proteins with putative function in transport process (i.e. those involved in the directed movement of substances into a living organism by a means of some agent such as a transporter or pore) (Additional file 1: Table S2, S3, S4). A total of 69 genes associated with the secondary metabolism showed significant differential expression (Additional file 1: Table S2, S3, S4). Among the induced genes, we found twelve Cytochrome P450 genes, whose expression profile changed during the early stage of infestation from being down- to up-regulated. The Cytochrome P450 belongs to a broad class of enzymes involved in a wide range of biosynthetic reactions. For instance, P450s act at different points of the phenylpropanoid biosynthetic pathway, as well as in the synthesis of plant allelochemicals, (e.g.: insect toxins, repellents or attractants) [37]. At 48 h, the expression of five genes coding for enzymes involved in polyamine metabolism was repressed, (a S-adenosyl-l-methionine synthase, two Arginine decarboxylases, a Spermidine synthase and an Ornithine decarboxylase). These molecules are an integral part of plant stress response, working as antioxidants, free radical scavengers and membrane stabilizers [38]. Additionally at 48 h, a total of 19 genes associated with the phenylpropanoids and alkaloids biosynthesis were downregulated.

### Array validation by qRT-PCR of defense-related genes

To validate the microarray results, the expression of nine differentially expressed genes was analyzed by Real Time-PCR. We selected genes annotated in different biological processes (biotic defense response, signaling transduction, phytohormone signaling and transcriptional regulation). The Additional file 2: Figures S1, S2 and S3 show the relative quantity (RQ) of each target gene in infested plants at each harvest time. The results were consistent to the microarray data, since the genes analysed displayed similar differential gene expression in response to aphids.

### Proteomic analysis of *M*. *euphorbiae*-infested tomato leaves

**Figure 5:**
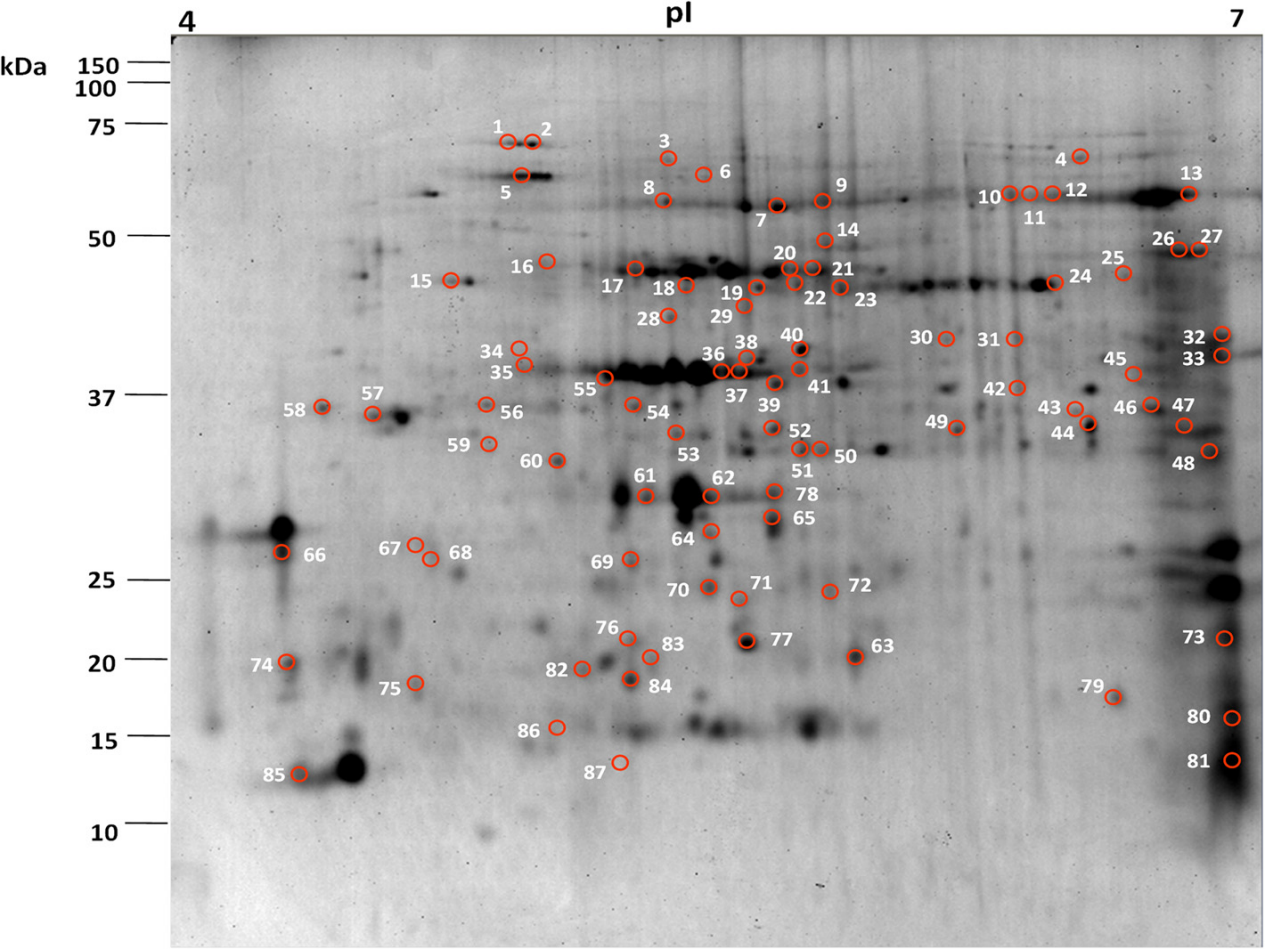
**2-DE proteomic map of tomato leaves from non-infested tomato plants.** Protein extracts were analyzed in first dimension (pH 4–7 linear IPG, 18 cm); second dimension was performed on a vertical slab (12%T) gel. Protein detection was achieved by using colloidal Coomassie staining. Numbering refers to differentially-represented protein spots in the *M*. *euphorbiae*-infested plants, which were then excised, digested and identified by MS procedures as reported in Additional file 1: Table S5.

**Figure 6:**
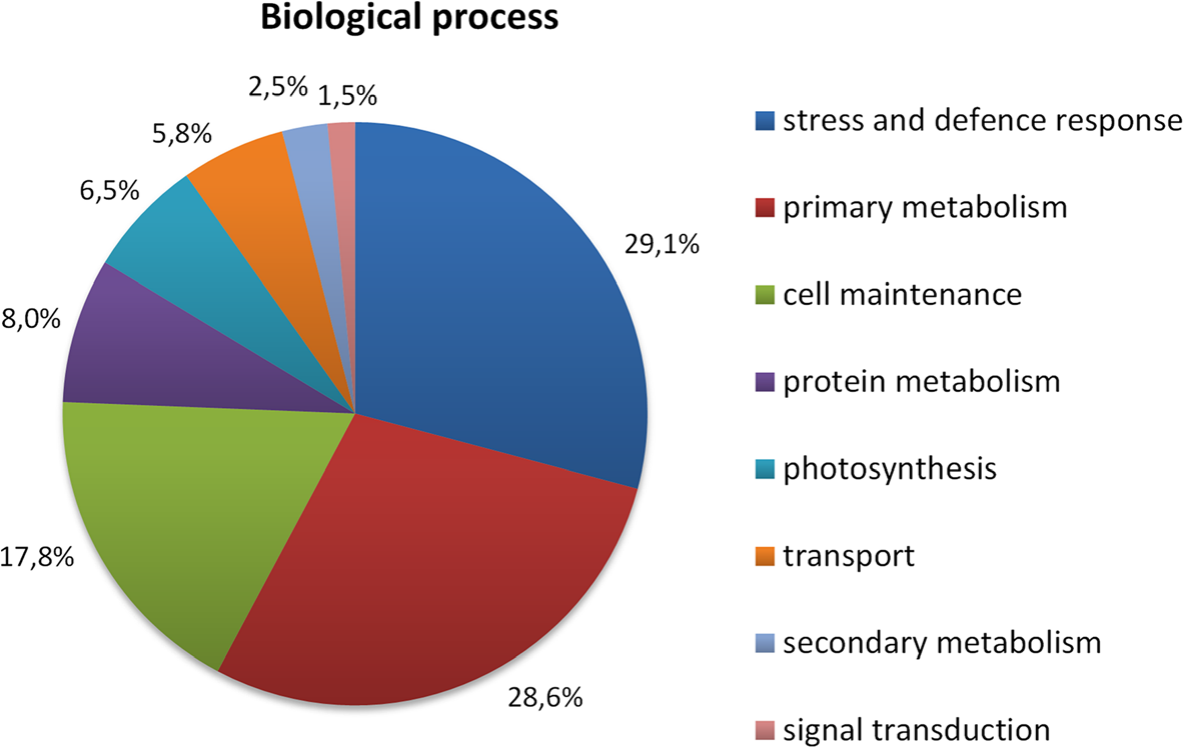
**Assignment of Gene Ontology (GO) terms for differentially-expressed proteins after *M*. *euphorbiae *infestation in tomato.** Components, such as Biological Process, are indicated. Individual GO categories can have multiple mappings. Percentages refer to the total categories annotated and not the total sequences annotated under each component.

We also carried out a proteomic analysis of tomato leaves after *M*. *euphorbiae* damage. The leaf tissue of control and infested plants at 48 hours following infestation was used for protein extraction. Proteins were subjected to 2-DE analysis and a representative Coomassie-stained gel from control leaves is shown in Figure 5. Peptide spots showing qualitative and statistically different quantitative differences between infested and control plants were further analyzed. Eighty-seven spots were selected as differentially expressed in tomato after aphid damage with a cut-off of a twofold change compared to the control. A database search with data from peptide mass fingerprinting using MALDI-TOF-MS experiments allowed the identification of the protein uniquely present in 45 spots; the remaining ones were analyzed by nanoLC-ESI-LIT-MS/MS, which identified 12 additional unique components. In the residual 30 spots, we detected multiple polypeptide species, which did not allow a quantitative evaluation of the protein expression level. The list of all the identified proteins is reported in Additional file 1: Table S5, together with the corresponding quantitative variations. The annotation of their protein coding genes indicated that the most represented biological process was “stress and defence response”, followed by “primary metabolism” (Figure 6). Among the differentially represented proteins after aphid attack, those involved in the photosynthesis included the oxygen-evolving enhancer protein 1 (OEE1) (spots 35, 36, 37, 41 and 83), oxygen-evolving enhancer protein 2 (OEE2) (spots 61, 62, 65 and 78), photosystem II oxygen-evolving complex protein 3 (spot 73), ATP synthase subunit beta (spots 7, 8 and 9), ATP synthase (spot 42 and 46) and cytochrome f (spot 31). A similar trend was also observed for enzymes of the photorespiration system, such as the RuBisCO activase (spots 17, 20, 21 and 59), RuBisCO decarboxylase small chain (spot 86), aminomethyltransferase (spot 26 and 27) and glycine/serine hydroxymethyltransferase (spot 11 and 13). As a result of concomitant multiple spot changes often with a negative quantitative trend, some of them showed variations that were suggestive of the occurrence of post-translational modifications. All proteins occurring in multiple spots have been already reported to be phosphorylated on other plant species (http://phosphat.mpimp-golm.mpg.de and http://www.p3db.org). A down-representation of proteins involved in carbohydrate metabolism, namely glycosyl hydrolase family 3 protein (spot 4) and triosephosphate isomerase (spots 44, 49 and 53), was also observed. Among the proteins related to the “transport” category, the mitochondrial outer membrane protein porin (VDAC) (spot 33) and ferredoxin-1, chloroplastic (spot 66 and 74) were up-regulated. Ferredoxin also participates in other reactions in the chloroplast (*e*.*g*. redox regulation) [39] and is strongly up-regulated after pathogen attack [40,41]. Many proteins correlated to defense and stress response were down-regulated in infested plants, except for the Nodulin-related protein (spot 67). Among the down-regulated proteins two were involved in oxidative stress, such as thioredoxin peroxidase 1 (spot 64) and oxidoreductase (spot 23). The category “protein metabolism” included proteins involved in translation, complex assembly, proteolysis and folding (spots 1, 2, 3, 6, 14, 15, 19, 28, 43 and 87). All these proteins, except for the chaperonin 20 (spots 50 and 51), were down-represented, suggesting that protein synthesis and secretion patterns are significantly affected in infested tomato. Finally, glycine-rich RNA-binding protein and RNA recognition motif (RRM)-containing protein were down-regulated, as also observed in rice leaf sheaths in response to infestation by the brown planthopper (*Nilaparvata lugens*) [42].

### Protein-mRNA comparative analysis

A correlation of transcriptomic and proteomic data was performed considering the genes that give rise to the differentially expressed transcripts or proteins. A first comparison, focusing on the functional categories in which the different protein species and transcripts were ranked, indicated a correspondence between the identified GO categories. For both “omics” studies, the functional category “defense and stress response” was the most represented. The match between genes and proteins was very limited, with only 3 mRNA-protein identities (Table 3). Correspondence between differentially expressed genes and proteins was only found considering the transcriptomic data at 48 h. It is therefore likely that factors such as the regulation of mRNA translation and post-translational processing seem to have a more relevant role than the time-lag between transcription and translation to account for the weak correlation between the “omics” data.

**Table 3:** List of common differentially-expressed transcripts and -represented proteins as obtained from microarray and combined 2-DE/MS studies

			**Fold change**			
**Description**	**Unigene**	**Spot**	**Proteomic**	**Transcriptomic**		
				**24 h**	**48 h**	**96 h**
Photosystem II oxygen-evolving complex protein 3	Solyc02g079950.2.1	73	14.4	-	−2.31	-
Plastid-lipid associated protein PAP	Solyc09g090330.2.1	55	0.2	-	2.40	-
Ribulose bisphosphate carboxylase small chain 1	Solyc02g063150.2.1	86	Off	-	−2.23	-

## Discussions

Our study generated extensive data on the expression of a large number of tomato genes and proteins during critical periods of infection with aphids and it provides the first insight into the dynamics of the response to *M*. *euphorbiae* attack. We analyzed plants at the early stage of infestation, before the development of visible symptoms, and interestingly, the biological processes and molecular pathways affected by aphid feeding were consistent with changes in other plant-aphid interactions [43-45]. However, in the present work a considerable number of tomato genes has been for the first time related to aphid response, thus providing new knowledge on the overlap and interaction between signal transduction pathways and defense response elicited by aphids in tomato.

Even if aphids are able to establish a long-lasting intimate interaction with plant cells, the tomato reaction was clearly variable during the course of the infestation. The number of differentially expressed genes considerably increased from 24 h to 48 h, reaching also the higher magnitude of expression, and declined at 96 h. This trend may be explained considering that a multifaceted defense response is costly for the plant, which should try to balance defense induction and impact on fitness traits following probing and establishment of a feeding site. Furthermore, in a compatible plant-insect interaction, it is likely that the reduced magnitude of the response at the last stage is the result of plant reprogramming the injured leaf to handle the progression of a successful infestation, along with an active aphid deception of tomato defense [46,47].

For a more integrative view of the tomato-aphid interaction, the transcript profiling was complemented by a proteomic study. We confidently identified 57 proteins that were differentially expressed after insect attack. These proteins belong to a set of biological processes that covered the functional groups of the transcriptional analysis. For instance, response to stress and alteration in photosynthesis were the most abundant categories. The little overlap between trancscriptomic and proteomic data is in accordance to other combined studies in plants [48,49]. A likely explanation is related to the features of the proteomic approach [50]. The set of protein spots identified as quantitatively altered by aphids should not be considered comprehensive and other differentially expressed proteins (e.g. transcriprion factors) may have not been detected in 2-D gels due to low concentration and poor solubility in our experimental conditions. Finally, these discrepancies can be due to the post-transcriptional and post-translational events that might be enhanced during tomato defense response to aphid damage [51].

**Figure 7:**
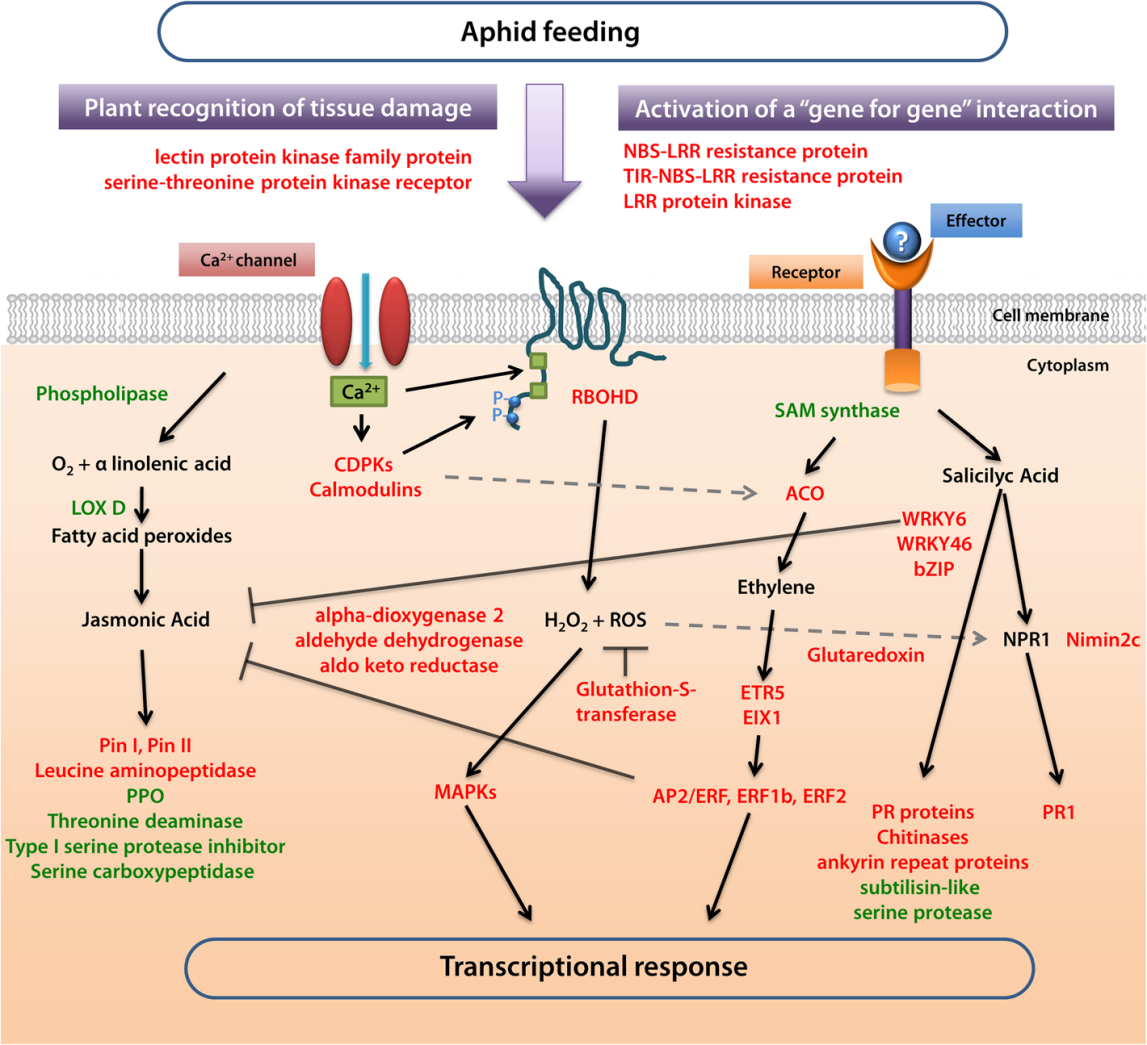
**A model summarizing the signaling events and molecular response in tomato following aphid attack.** Selected up-regulated genes are in red color, while down-regulated genes are in green. Black lines with arrows indicate activation of enzymatic activities, induction or accumulation of compounds. Grey dashed lines represent indirect positive interactions. Black blunted lines represent inhibitory associations. See text for details.

Based on the transcriptomic and the proteomic data, we propose a model to depict the main components involved in aphid response in tomato (Figure 7). Our data indicated that, at the early stage of infestation, *M*. *euphorbiae* triggers the induction of receptors (e.g. lectin kinase receptors) responsive to wounding and to oligogalacturonic acids signals [52], but also of genes coding for receptors (e.g. RLKs and LRR-RLKs) that play a central role in signaling following recognition of fungal pathogens [53]. Among others, aphid attack induced a strong activation of a NBS-LRR gene. These proteins detect the presence of disease-causing bacteria, viruses, or fungi by recognizing specific pathogen- or plant-derived effectors [54]. The data strongly support a model in which plants perceive aphids due to tissue damage and to a gene-for-gene recognition of aphid-derived elicitors [2]. Currently, it is not known if *M*. *euphorbiae*, as other aphids, delivers effectors [4], yet the concurrent activation of different receptors would explain why *M*. *euphorbiae* elicits in tomato a signaling cascade overlapping wounding and pathogen response.

Tomato should perceive aphids essentially through cell membrane receptors and subsequently, the signal transduction exploits various cellular messengers, principally ROS, calcium and stress hormones [14,44,55]. Both transcriptomic and proteomic data indicated differences in genes related to ROS responsive or metabolizing systems (e.g. metallothionein, l-ascorbate oxidase homolog, Respiratory Burst Oxidase-like Protein). In addition, the results underlined the down-regulation of genes associated with the polyamine metabolism, molecules involved in various physiological events such as development, senescence and stress responses including oxidative stresses [56]. Down-regulation of all those genes following potato aphid infestation would also allow the plant to maintain high H_2_O_2_ levels that can damage the insect midgut [11]. On the other hand, the induction of ROS detoxifying enzymes (e.g. glutaredoxin, glutathione-s-transferase, aldo-keto reductase, α-DOX), represents an effort of tomato cells to keep ROS levels below toxicity. Among these genes, peroxidases showed the higher expression levels, as also reported for the barley-*Diuraphis noxia* interaction [45], showing that these enzymes are an important component of the plant reaction to aphids. The tomato response includes genes coding for calcium-dependent kinases (CDPK1, CDPK2, CDPK3) and calcium-binding proteins (Calmodulin-binding protein, Calmodulin-like protein), further confirming the involvement of this ubiquitous intracellular messenger in signal transduction in tomato [10,16]. In addition, the concurrent up- and down-regulation of genes coding for calcium binding proteins is consistent with an elaborate role of calcium in plant-aphid interaction, since its concentration is at the core of the molecular sabotage that aphids carry out to avoid sieve-plate occlusion [57]. Calcium and ROS signaling should be associated through the regulation of RBOHD expression [58]. NADPH oxidases are key plasmalemma-bound enzymes for stress-induced ROS production in plants [58,59]. The presence of EF-hand calcium-binding motifs in the up-regulated tomato RBOH protein suggests that aphid-induced Ca^2+^ influxes should affect NADPH oxidase activity through the phosphorylation of the N-terminal region of the protein by CDPKs [60]. The recognition of aphid feeding by tomato receptors triggers the defense reaction through an interplay of different stress-related phytohormones. What is the contribution of JA and SA to plant defenses against aphids, and which of these hormones has the predominant role is not yet clear. Growing evidence underlines the importance of JA-dependent defenses to hamper aphid infestation [17,61]. For instance, jasmonate application improves aphid resistance in different plant species [11,62,63]. Furthermore, aphid population growth is boosted or suppressed in *Arabidopsis* mutants impaired or enhanced in the JA pathway, respectively [64,65]. Different studies also emphasize the contribution of SA to plant defenses against aphids and it has also been proposed that the SA pathway has a significant role in aphid-resistant genotypes, primarily by promoting antibiosis or repellence [9,66,67]. While SA does not seem to be as important as JA in *Arabidopsis*, only recently, it has been observed that the JA-dependent responses do not significantly contribute to antibiotic defenses against aphids in tomato, while basal resistance is dependent on SA accumulation [68]. Our data showed that several genes responding to SA were up-regulated, whereas a lower number of genes associated to JA biosynthesis, wounding as well as JA-regulated genes were either repressed or showed mild changes in their expression level. Our work stressed the prevailing involvement of SA during the establishment of a tomato-aphid compatible interaction, along with a possible antagonistic crosstalk between the SA- and JA-signaling pathways. Under this perspective, the overexpression of genes involved in ethylene synthesis and signaling (ACO, ERF1b, ERF2, AP2/ERF transcription factor, ETR5) should play a dual role. On one hand it has a synergistic effect by additively improving induced responses [69,70]. In parallel, the overexpression of Ethylene Responsive Factor genes should also restrain the JA-pathway [71,72]. This is also supported by the overexpression of harpin-related genes. It has been proposed that stimulation of plants by harpin separates the roles of Et and JA in plant-aphid defense, by promoting Et and SA and suppressing JA signaling [73]. Finally, the induction of genes involved in brassinosteroid synthesis and signaling indicates that this hormone is also involved in the tomato defense response to aphids, most likely by contributing to antagonize the JA pathway [74]. Taken together, the data imply that tomato uses a composite interplay of plant hormones to modulate a JA-independent inducible defense in a compatible aphid interaction [75].

The tomato transcriptional reconfiguration relies in a number of TFs, and a pivotal role is played by WRKY proteins. As some of these proteins mediate the cross-talk between JA-mediated and SA-mediated signals during plant defense, it is reasonable to speculate that the WRKY genes that are continuously overexpressed during the aphid infestation, are involved in the antagonist interaction between SA and JA, as reported for the WRKY70 in *Arabidopsis*[22,76].

Although the number of differentially expressed genes and proteins that can directly affect aphid performance was low in percentage, the tomato response includes changes in cell structure and plant metabolism that can successfully limit aphid infestation, as also observed in other interactions [12,13,45,55]. It is likely that the downregulation of genes involved in the catabolism of cell wall components is a cost-effective strengthening strategy of the cell wall structure. On the other hand, the down-regulation of the Glucan endo-1 3-beta-glucosidase 5 (Gns5), which plays a key role in callose decomposition, may be indicative of the plant’s effort to favor sieve tube occlusions, preventing phloem ingestion. Callose deposition is essential to occlude injured sieve elements and avoid sap loss [77]. Hence, the observed pattern of expression suggests that the tomato defense strategy will provide a barrier to the insect stylet and puncturing and it will also limit food supplies to aphids.

Transcriptomic and proteomic data showed a consistent reduction in the category ‘photosynthesis’. The transcriptional downregulation of photosynthetic-related genes appears to be a kind of universal adaptive response of plants to biotic stress, which may be compensated by a slower turnover of many photosynthetic proteins [78]. Our data showed that downregulation is seen also at the protein level, indicating that in tomato the induction of a multi-component defense response to aphids requires repression of other cellular functions to ensure metabolic balance [78]. However, protein profiling revealed an increased accumulation of some photosynthesis-related proteins, such as two proteins of PSII system (OEE1 e OEE2) and the Ferredoxin protein. This up-regulation may be related to the role of these proteins in defense, rather than a response of photosynthesis *per se*. The OEE2 is a downstream protein of the AtGRP-3/WAK1 signaling pathway complex involved in the SA-dependent defense response in *Arabidopsis*[79]. Similarly, OEE1 exhibits properties and enzyme-modulating activities of a thioredoxin, and it may act protecting cells from the oxygen radicals formed in response to abiotic and biotic stress [80]. Other down-regulated genes and proteins are involved in plant metabolism. Aphids are able to alter the source–sink relationships into the plant by the ingestion of great volumes of phloem sap to fulfill their nutritional requirements [81]. Hence, the negative regulation of genes associated with the primary metabolism may be a strategy adopted by tomato to limit the plant resources assimilation from *M*. *euphorbiae*. This is also in accordance with the down regulation of different genes involved in carbohydrate and water transport. For instance, genes coding for Aquaporin-like proteins were down-regulated, as in *Citrus sinensis* plants after *H*. *coagulata* feeding [82]. On the other hand, different genes associated with amino acid and nitrogen translocation were found to be up-regulated. *M*. *euphorbiae* is expected to modify nitrogen allocation in tomato plants by competing with plant sinks and altering the amino acid composition of the phloem sap [83,84]. The observed deregulation confirms that aphids are able to extensively manipulate plant physiology in relation to their nutrition status [1,85]. From a metabolic perspective, our data indicated that tomato response presumably allows energy reallocation to prioritize specific defense responses, while modulating other important functions to indirectly reduce aphid performance.

## Conclusion

Despite the relevant economic impact, not much is known about the molecular recognition and response of tomato plants, and more generally of Solanaceae crops, to aphids. Our study provided a detailed overview of the transcriptomic and proteomic responses of tomato to aphids and led to a more comprehensive understanding of the signaling events and the defense dynamics. Different molecular cues, including those associated to tissue damage and elicitor recognition, lead to a complex, dynamic pattern of expression, in which distinct groups of similarly behaving transcripts were observed. Early events of the response support a gene-for-gene interaction and sensing of a wound-induced damage. The Gene Ontology categories of the identified genes and proteins indicated that the local response is characterized by an increased oxidative stress accompanied by the production of proteins involved in the detoxification of oxygen radicals. Aphids elicit a defense reaction that involves different hormones, with the SA-signaling pathway and stress-responsive SA-dependent genes playing a dominant role. The wound-inducible JA pathway was not strikingly affected, although some JA-dependent genes coding for anti-nutritive proteins were up- or down-regulated. Finally, tomato response is characterized by a reduced investment in photosynthetic proteins and a modification of the expression of various cell wall-related genes.

The identification of genes involved in aphid defense, provides a reference line for the screening of tomato genomic resources, eventually impacting other economically important Solanaceae crops. In the future, targeted functional studies should follow to elucidate the role of the here presented genes in the tomato defense, essential for the development of rational strategies to enhance a durable broad-spectrum resistance to aphids in tomato.

## Methods

### Biological material

Four weeks old tomato plants (*Solanum lycopersicum* L. ‘Microtom’), grown in insect-proof cages under a 16 h day cycle at a temperature of 25 ± 1°C, were used as host. *M*. *euphorbiae* (Thomas) was obtained from an insect culture maintained at the Dipartimento di Agraria, Università di Napoli Federico II, and it was reared on tomato plants ‘M82’. Ten 2^nd^/3^rd^ instars were gently transferred onto the abaxial surface of sub-terminal lateral leaves with the help of a paintbrush. Aphids were confined to specific leaves on the plant and their number controlled daily. Uninfested plants were grown under the same conditions. To monitor changes in host gene expression, leaves from infested plants and synchronous aphid-free controls were harvested at 24, 48 and 96 h after infestation. Aphids were manually removed from leaves and the tissues were frozen in liquid nitrogen for subsequent analysis, carried out on three biological replicates per time point. Leaves of a single replicate were pooled to reduce noise arising from biological variation.

### Microarray analysis

#### RNA purification, labeling and oligonucleotide microarray hybridization

Approximately 200 mg of leaf tissue were ground to fine powder in liquid nitrogen and homogenized in Qiazol solution (Qiagen). RNA was isolated using the RNeasy Plus Mini kit (Qiagen), according to manufacturer’s protocol. RNA samples were analyzed quantitatively and qualitatively with a NanoDrop ND-1000 UV–vis Spectrophotometer (NanoDrop Technologies) and by a Bioanalyzer (Agilent Technologies), respectively. Only samples with an RNA Integrity Number (R.I.N.) >8 were used for RNA labeling. Total RNA was amplified in the presence of cyanine-3/cyanine-5 labeled CTP using the Agilent low RNA Input Fluorescent Linear Amplification kit (Agilent Technologies), according to manufacturer’s instructions. After labeling, reactions were purified using the columns of the Qiagen’s RNeasy kit. The quality of labeled targets was determined by calculating the amount of cRNA concentration (ng/μl), Cyanine 3 or cyanine 5 dye concentration (pmol/μl) and RNA absorbance ratio (260/280 nm) using a NanoDrop Spectrophotometer. The specific activity was calculated using the formula: (Concentration of Cy3 or Cy5) / (Concentration of cRNA) * 1000 = pmol Cy3 or Cy5 per μg cRNA. Only samples with a specific activity ≥8 were used for hybridization. Equal amounts of cRNAs from control and from infested plants were mixed together and hybridized to the microarray in a hybridization oven at 65°C for 17 hours with rotation speed set at 10 rpm.

Gene expression profiling was performed using the Tomato Gene Expression array (4x44k) (Agilent Technologies). This array contains over 44000 probes, representing more than 21,000 tomato transcripts. After hybridization, slides were washed with Gene Expression Wash buffer 1 for 1 minute at room temperature, and Gene Expression Wash buffer 2 for 1 minute at 37°C. Finally, arrays were treated with the Stabilization and Drying Solution (Agilent Technologies) for 30 seconds at room temperature. Immediately after washing, slides were scanned with the Agilent Dual Laser Microarray Scanner and image data were read out and processed by the Feature Extraction v. 10 software (Agilent Technologies).

#### Data analysis

The GeneSpring® 10 (Agilent Technologies) software was used to process the microarray data and to associate sample information. Statistical analysis was performed using background-corrected mean signal intensities from each dye channel. Microarray data were normalized using intensity-dependent global normalization (LOWESS). Statistical testing of differential expression was performed using the Benjamini-Hochberg False Discovery Rate method with a cut off (*p*-value) <0.05. Of the significantly differentially expressed annotated probes, only those with greater than 2-fold increase or 2-fold decrease in expression compared to the controls were used for further analysis. As the array was designed from ESTs before a reference tomato genome sequence was available, for each differentially expressed probe, a similarity analysis was conducted with blastN against SGN Tomato Unigene database (http://solgenomics.net/). We used an e-value threshold of 1^e-10^ to reduce redundancy on the array as well as possible imperfect probe matches. The comparative analysis was performed against a combination of all Tomato Unigenes, BACs, and BAC-end sequences predicted by the ITAG (International Tomato Annotation Group) official annotations, on the SL2.40 tomato genome build. Briefly, when possible, we located each probe on the tomato genome and associated each probe to a transcript of a single gene. Functional annotation of the differentially expressed genes was performed using the Blast2GO software [90] at the default parameters, followed by manual curation.

### Microarray validation by real-time quantitative PCR analyses

Two μg total RNA were reverse transcribed by using a SuperScript II Reverse Transcriptase (Invitrogen), following the manufacturer’s protocols. To control cDNA synthesis and PCR efficiency, the amplification of the constitutive gene coding for the Elongation Factor 1-a (EF1-a) was carried out according to already published procedures [86]. Real-time PCRs were performed as described [87]. The primers and the size of the expected amplicons are indicated in the Additional file 1: Table S1. For each target, reactions were performed in triplicate and experiments were carried out on three replicates for treatment. The relative quantification of the gene expression and its statistical test was conducted as previously described [86].

### Proteomic analysis

Proteins were isolated from leaves and resolved and scanned in 2D gels as described [88]. Image analysis was performed using the PDQuest software (Bio-Rad). Spot detection and matching between gels were performed automatically, followed by manual verification. Protein spots were annotated only if detectable in all gels. After normalization of the spot densities against the whole-gel densities, the percentage volume of each spot was averaged for nine different (three replicates of three biological samples) gels and Student’s t-test analysis (*p* <0.05) was performed to find out significant protein fold changes between aphid-challenged and control plants. A two-fold change in normalized spot densities was considered indicative of a differentially-expressed protein. For statistical analysis, data were analyzed by using the SPSS software (IBM) through missing value imputation via K-nearest neighbours analysis, followed by log-transformation of the imputed data and comparison of control and treated values to evaluate corresponding variance (ANOVA), with a non-linear mixed-effects model.

### Protein digestion and MS analysis

Spots from 2-DE were manually excised, triturated, *in*-*gel* reduced, S-alkylated and digested with trypsin, as previously reported [88]. Protein digests were subjected to a desalting/concentration step on microZipTipC_18_ pipette tips (Millipore Corp., Bedford, MA, USA) before MALDI-TOF-MS and/or nanoLC-ESI-LIT-MS/MS analysis. During MALDI-TOF peptide mass fingerprinting (PMF) experiments, peptide mixtures were loaded on the instrument target together with α-cyano-4-hydroxycinnamic acid as matrix, using the dried droplet technique. Samples were analysed with a Voyager-DE PRO mass spectrometer (AB Sciex, USA). Peptide mass spectra were acquired in reflectron mode over a mass range of 800–4000 Da, by averaging 50–300 laser shots, and manually inspected to get the corresponding peak lists; internal mass calibration was performed with peptides derived from trypsin autoproteolysis. Data were elaborated using the DataExplorer 5.1 software (AB Sciex). Peptide mixtures were also analyzed by nanoLC-ESI-LIT-MS/MS using a LTQ XL mass spectrometer (Thermo, USA) equipped with Proxeon nanospray source connected to an Easy-nanoLC (Proxeon, Denmark) [89]. Peptide mixtures were separated on an Easy C_18_ column (100 x 0.075 mm, 3 μm) (Proxeon) using a gradient of acetonitrile containing 0.1% formic acid in aqueous 0.1% formic acid; acetonitrile ramped from 5% to 35% over 24 min, at a flow rate of 300 nL/min. Spectra were acquired in the range *m*/*z* 300–1800. Acquisition was controlled by a data-dependent product ion scanning procedure over the three most abundant ions, enabling dynamic exclusion (repeat count 1 and exclusion duration 1 min). The mass isolation window and collision energy were set to *m*/*z* 3 and 35%, respectively.

### Protein identification

The MASCOT software package version 2.2.06 (Matrix Science, UK) was used to identify proteins present in gel spots from a tomato Unigene database (http://solgenomics.net/) (SGN 2009, 68026 sequences) and/or an updated plant non-redundant sequence database (NCBI nr 2009/04, 654658 sequences). Identified SGN entries were associated with corresponding plant proteins by using the BLAST algorithm (http://blast.ncbi.nlm.nih.gov/). In particular, MALDI-TOF PMF data were searched using a mass tolerance value of 40–80 ppm; nanoLC-ESI-LIT-MS/MS data were searched by using a mass tolerance value of 2 Da for precursor ion and 0.8 Da for MS/MS fragments. In both cases, searching was performed using trypsin as proteolytic enzyme, a missed cleavages maximum value of 2 and Cys carbamidomethylation and Met oxidation as fixed and variable modification, respectively. MALDI-TOF PMF candidates with a cumulative MASCOT score > 83 or nanoLC-ESI-LIT-MS/MS candidates with more than 2 assigned unique peptides with an individual MASCOT score > 25, both corresponding to *p* < 0.05 for a significant identification, were further evaluated by the comparison with their calculated mass and pI values, using the experimental values obtained from 2-DE. Each protein identification was verified manually. Protein functional analysis was performed using Blast2GO [90].

## Abbreviations

2-DE: Two-dimensional gel electrophoresis; ABA: Abscissic acid; CDPK: Calcium dependent protein kinase; Et: Ethylene; GO: Gene ontology; JA: Jasmonic acid; LRR: Leucin rich repeat; MALDI: Matrix-assisted laser desorption/ionization; MS: Mass spectrometry; NADPH: Reduced nicotinamide adenine dinucleotide phosphate; NBS: Nucleotide binding site; PPO: Polyphenol oxidase; PPR: Pentatricopeptide repeat-containing; PR: Pathogenesis related; RLK: Receptor like kinase; ROS: Reactive oxygen specie(s); SA: Salicilic acid; SAR: Systemic acquired resistance; TOF: Time of flight

## Competing interests

The authors declare that they have no competing interests.

## Authors’ contributions

VC performed the microarray and the gene expression analysis, analyzed the data and drafted the paper, MC contributed to the microarray experiments and the functional annotation, MR performed the 2D-Electrophoresis, CD carried out the aphid infestation, CDA and GR executed the analysis of the proteomic spots, RM provided assistance for the microarray experiments, AS analyzed the proteomic data and contributed to the writing of the manuscript, FP reviewed the manuscript, RR participated in the study design and reviewed the manuscript, GC designed the study, analyzed the data and wrote the manuscript. All authors read and approved the manuscript.

## Supplementary Material

Additional file 1: Table S1. Primers used for the expression study and their main features. Table S2: Differentially expressed genes 24 h after infestation with *M. euporbiae*. Table S3: Differentially expressed genes 48 h after infestation with *M. euphorbiae*. Table S4: Differentially expressed genes 96 h after infestation with M. euphorbiae. Table S5. Protein species showing quantitative changes during aphid infestation as identified by combined 2-DE and MS procedures. Click here for file

Additional file 2: Figure S1. Microarray validation and concordance with Real Time results (A) Relative gene expression analysis 24 h following aphid infestation. The graph displays the relative quantity (RQ) of each target gene in infested plants relative to the calibrator control plants. Asterisks indicate that the 2-∆Ct values were significantly different from the calibrator (p < 0.01; Student’s t-test). (B) The graph displays the concordance between microarray fold change and Real Time RQ values on a linear scale. Figure S2: Microarray validation and concordance with Real Time results (C) Relative gene expression analysis 48 h following aphid infestation. The graph displays the relative quantity (RQ) of each target gene in infested plants relative to the calibrator control plants. Asterisks indicate that the 2-∆Ct values were significantly different from the calibrator (p < 0.01; Student’s t-test). (D) The graph displays the concordance between microarray fold change and Real Time RQ values on a linear scale. Figure S3: Microarray validation and concordance with Real Time results (E) Relative gene expression analysis 96 h following aphid infestation. The graph displays the relative quantity (RQ) of each target gene in infested plants relative to the calibrator control plants. Asterisks indicate that the 2-∆Ct values were significantly different from the calibrator (p < 0.01; Student’s t-test). (F) The graph displays the concordance between microarray fold change and Real Time RQ values on a linear scale. Click here for file
