# Supplementary material for: Transcriptomic and proteomic analysis of a compatible tomato-aphid interaction reveals a predominant salicylic acid-dependent plant response
Source: BMC Genomics. 2013 Jul 29;14:515. doi: 10.1186/1471-2164-14-515 (PMC3733717; doi:10.1186/1471-2164-14-515)
Supplement: Additional file 2: Figure S1. — Microarray validation and concordance with Real Time results (A) Relative gene expression analysis 24 h following aphid infestation. The graph displays the relative quantity (RQ) of each target gene in infested plants relative to the calibrator control plants. Asterisks indicate that the 2-∆Ct values were significantly different from the calibrator (p < 0.01; Student’s t-test). (B) The graph displays the concordance between microarray fold change and Real Time RQ values on a linear scale. Figure S2: Microarray validation and concordance with Real Time results (C) Relative gene expression analysis 48 h following aphid infestation. The graph displays the relative quantity (RQ) of each target gene in infested plants relative to the calibrator control plants. Asterisks indicate that the 2-∆Ct values were significantly different from the calibrator (p < 0.01; Student’s t-test). (D) The graph displays the concordance between microarray fold change and Real Time RQ values on a linear scale. Figure S3: Microarray validation and concordance with Real Time results (E) Relative gene expression analysis 96 h following aphid infestation. The graph displays the relative quantity (RQ) of each target gene in infested plants relative to the calibrator control plants. Asterisks indicate that the 2-∆Ct values were significantly different from the calibrator (p < 0.01; Student’s t-test). (F) The graph displays the concordance between microarray fold change and Real Time RQ values on a linear scale. [file 1471-2164-14-515-S2.pptx]

## Slide 1
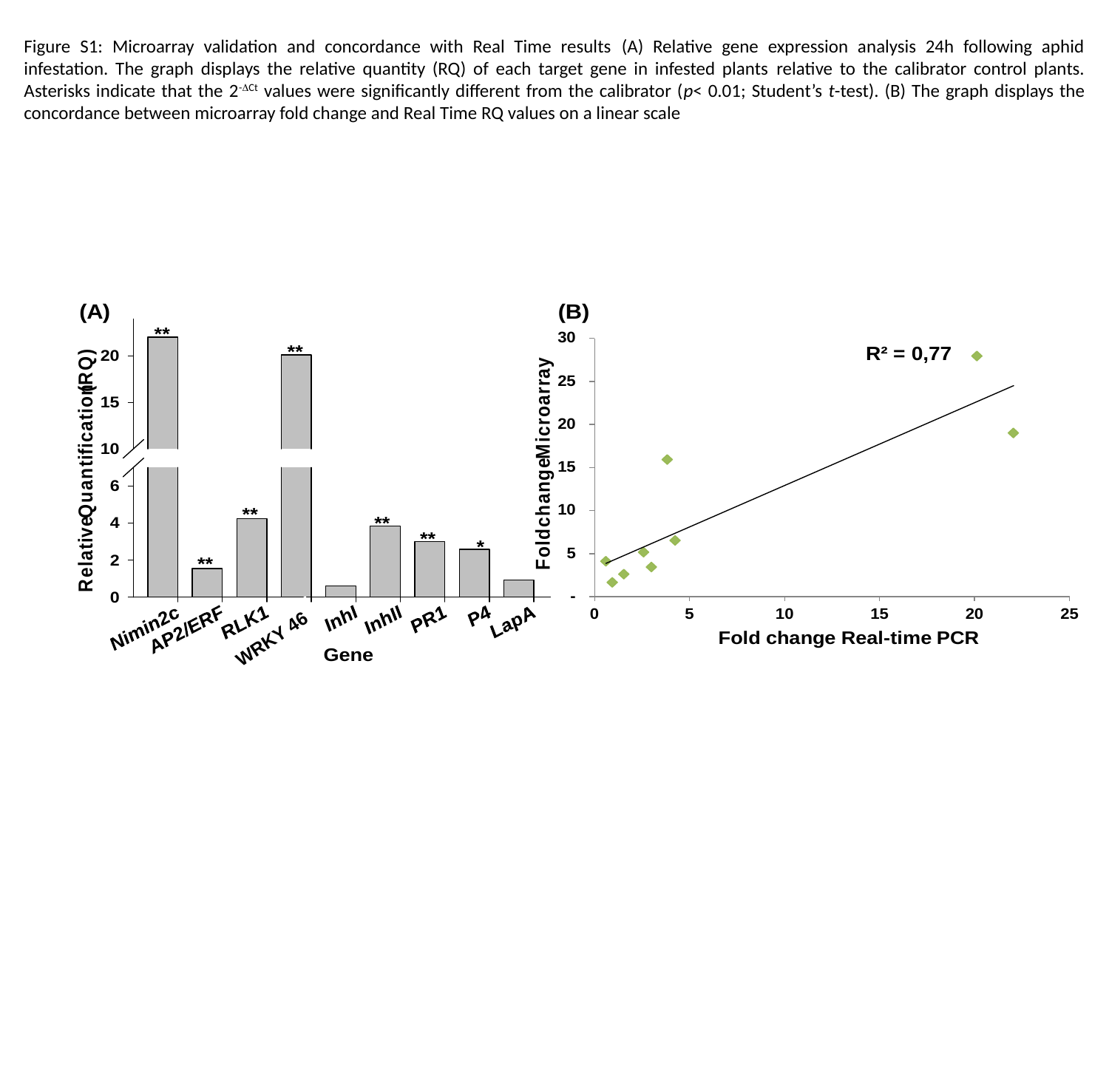

Figure S1: Microarray validation and concordance with Real Time results (A) Relative gene expression analysis 24h following aphid infestation. The graph displays the relative quantity (RQ) of each target gene in infested plants relative to the calibrator control plants. Asterisks indicate that the 2-ΔCt values were significantly different from the calibrator (p< 0.01; Student’s t-test). (B) The graph displays the concordance between microarray fold change and Real Time RQ values on a linear scale
WRKY 46

## Slide 2
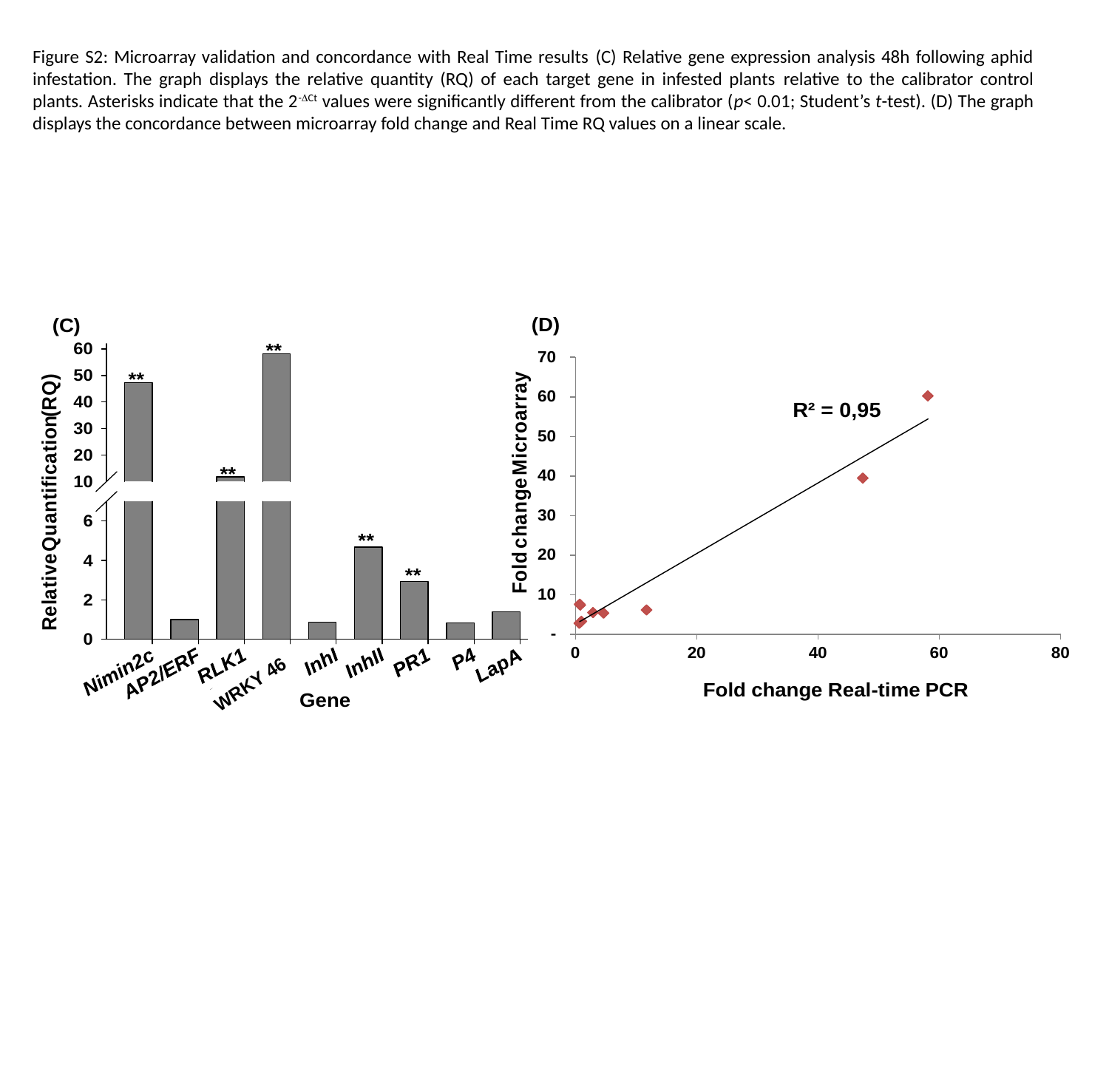

Figure S2: Microarray validation and concordance with Real Time results (C) Relative gene expression analysis 48h following aphid infestation. The graph displays the relative quantity (RQ) of each target gene in infested plants relative to the calibrator control plants. Asterisks indicate that the 2-ΔCt values were significantly different from the calibrator (p< 0.01; Student’s t-test). (D) The graph displays the concordance between microarray fold change and Real Time RQ values on a linear scale.
WRKY 46

## Slide 3
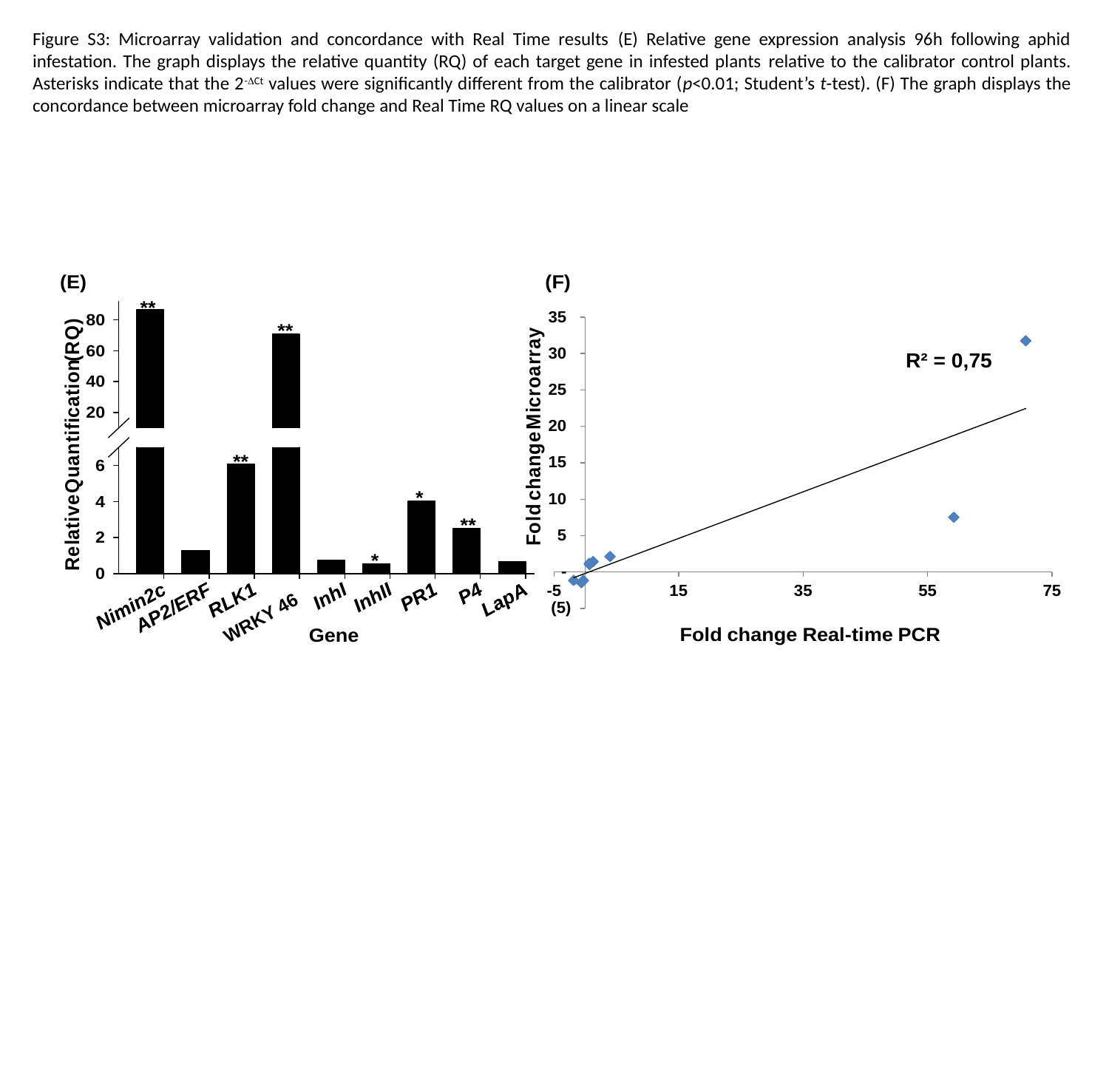

Figure S3: Microarray validation and concordance with Real Time results (E) Relative gene expression analysis 96h following aphid infestation. The graph displays the relative quantity (RQ) of each target gene in infested plants relative to the calibrator control plants. Asterisks indicate that the 2-ΔCt values were significantly different from the calibrator (p<0.01; Student’s t-test). (F) The graph displays the concordance between microarray fold change and Real Time RQ values on a linear scale
WRKY 46
